# Mode of the Interaction of Efflux Inhibitor Phenylalanyl-arginyl-β-naphtylamide with Bacterial Cells

**DOI:** 10.3390/biomedicines12061324

**Published:** 2024-06-14

**Authors:** Sandra Sakalauskaitė, Valeryia Mikalayeva, Simona Sutkuvienė, Rimantas Daugelavičius

**Affiliations:** 1 Department of Biochemistry, Vytautas Magnus University, 44248 Kaunas, Lithuania; valeryia.mikalayeva@lsmu.lt (V.M.); simona.sutkuviene@vdu.lt (S.S.); 2 Laboratory of Immunology, Department of Immunology and Allergology, Lithuanian University of Health Sciences, 50161 Kaunas, Lithuania; 3 Institute of Cardiology, Lithuanian University of Health Sciences, 50162 Kaunas, Lithuania; 4 Department of Biochemistry, Lithuanian University of Health Sciences, 50161 Kaunas, Lithuania; 5 Research Institute of Natural and Technological Sciences, Vytautas Magnus University, 44248 Kaunas, Lithuania

**Keywords:** efflux pump inhibitor, ion-selective electrode, LPS, phenylalanyl-arginyl-β-naphthylamide, Polymyxin B, *S.* Typhimurium

## Abstract

An increased efflux activity is one of the major reasons for bacterial antibiotic resistance. The usage of efflux pump inhibitors could be a promising approach to restoring the activity of inefficient antibiotics. The interaction of the RND family efflux pump inhibitor phenylalanyl-arginyl-β-naphthylamide (PAβN) with *Salmonella enterica* ser. Typhimurium cells was assayed using traditional microbiological techniques and a novel PAβN-selective electrode. Monitoring the PAβN concentration in the medium using the electrode enabled the real-time measurements of this compound’s interaction with bacterial cells. We showed that *S.* Typhimurium cells accumulate a high amount of PAβN because of its high affinity to lipopolysaccharides (LPSs), the major constituent of the outer layer of the outer membrane, and does not affect the functioning of the plasma membrane. EDTA enhanced the binding of PAβN to *S.* Typhimurium cells and the purified *E. coli *LPSs, but the energization of the cells by glucose does not affect the cell-bound amount of this inhibitor. Polycationic antibiotic Polymyxin B released both the cells accumulated and the suspended LPS-bound PAβN.

## 1. Introduction

The resistance to antibiotics is one of the top global public health and development threats. In addition to a low outer membrane (OM) permeability to lipophilic compounds and a high activity of periplasmic β-lactamases, highly expressed efflux pumps are one of the major causes of antibiotic resistance in *Salmonella enterica* ser. Typhimurium [1,2]. It is known that efflux pumps also contribute to bacterial virulence [3] and biofilm formation [4]. Therefore, an inhibition of the efflux could be a promising strategy to restore the potency of antibacterial agents [5]. The main and clinically most important efflux pumps in Gram-negative bacteria belong to the resistance–nodulation–division (RND) superfamily [6] and phenylalanyl-arginyl-β-naphthylamide (PAβN) is one of the most popular inhibitors of these devices [7,8]. Although PAβN is the best thoroughly studied efflux inhibitor, the mechanism of its action still needs to be fully understood. Two opinions prevail: PAβN is the OM permeabilizer [9] and/or it changes the conformation of the PM-located lipophilic pocket of the AcrB pump, decreasing its affinity to antibiotics [10].

The most common method for monitoring the efflux activity in bacteria is the fluorimetric estimation of the cell-bound ethidium, which is a substrate of many efflux pumps [11]. The higher levels of the accumulated ethidium inside the cells lead to increased fluorescence, indicating that the efflux is reduced and the indicator compound is not removed from the cytosol [12]. However, ethidium binds to the cell DNA and it is difficult to determine the exact concentration of this indicator accumulated in the cytosol. Therefore, the risk of an underestimated efflux level remains [11].

The efficiency of efflux pumps can be monitored using lipophilic cation-selective electrodes [13,14]. These cations are indicators of membrane voltage (transmembrane difference of electrical potential, Δψ) in mitochondria and bacteria. The most popular representative of these ions is tetraphenylphosphonium (TPP^+^). The Δψ (negative inside) bacteria accumulate TPP^+^ and the concentration of this cation inside the cells can be more than 10^3^ times higher than in the incubation medium [15]. However, despite a high Δψ on the PM, this lipophilic cation is not able to equilibrate across the envelope of Gram-negative bacteria because of the OM barrier. By measuring the interaction of TPP^+^ with Gram-negative bacteria, it is also possible to detect the changes in the permeability of the OM barrier [15,16]. In 1986, Midgley [17] showed that the level of TPP^+^ accumulation in bacteria depends also on the efficiency of pumps extruding this indicator cation back to the incubation medium. Further studies revealed that lipophilic cations are the most universal substrates of the efflux pumps in prokaryotic [13,14,18,19] and eukaryotic [19,20] cells. The monitoring of TPP^+^ accumulation in bacterial cells in the presence and absence of PAβN allows us to evaluate the totalefflux activity [21].

It is known [7] that PAβN interacts with a hydrophobic pocket of AcrB or analogous pumps in the bacterial PM, but it is not clear why this compound is so effective as an inhibitor, and how PAβN gets into the place of action. Based on our experience in the development of TPP^+^, phenyldicarbaundecaborane [15], and ethidium-selective [21] electrodes, we have constructed an electrode that is selective to PAβN. This device allowed us to assess the affinity of the *S*. Typhimurium cells to PAβN. In the current study, we demonstrated that *S*. Typhimurium cells bind a high amount of PAβN because of the affinity of this inhibitor to LPSs in the OM barrier. The high affinity of PAβN to bacterial LPSs and the accumulation of this inhibitor in the pump neighborhood could be an important factor in strengthening efflux inhibition in Gram-negative bacteria.

## 2. Materials and Methods

### 2.1. Bacterial Strains and Chemicals

*Salmonella enterica* ser. Typhimurium strain SL1344 and ∆acrB were obtained from Prof. Séamus Fanning (Institute of Food and Health, University College Dublin, Ireland). Phenylalanyl-arginyl-β-naphthylamide (PAβN) dihydrochloride, lipopolysaccharide (LPS, serotype *Escherichia coli* O11: B4, phenol extracted), polymyxin B (PMB) sulfate, Luria–Bertani broth (LB), Na_2_S_2_O_4_, chloramphenicol (Cm), tungstosilicic acid hydrate, poly(vinyl chloride) (PVC, average Mw ~80,000), and dioctyl phthalate were obtained from Sigma-Aldrich (Seelze, Germany). Ethylenediaminetetraacetic acid (EDTA), glucose, and HCl were obtained from Sharlau (Barcelona, Spain). Tris(hydroxymethyl)-aminomethane (Tris) and tetracycline (Tet) were obtained from Roth (Karlsruhe, Germany).

### 2.2. Conditions of Bacterial Growth and Determination of Minimal Inhibitory Concentration (MIC) of Antimicrobial Compounds

Overnight culture of *S*. Typhimurium cells was diluted 1:50 in fresh LB medium and grown with aeration at 37 °C to OD_600_ of 0.8. The cells were collected with centrifugation at 3000× *g* (Allegra TM64R; Beckman Coultier Inc., Fullerton, CA, USA) at 4 °C for 10 min. Pelleted cells were resuspended in 100 mM Tris/HCl (pH 8.0) buffer to obtain 1 mL of concentrated suspension (~2 × 10^11^ cells/mL), kept on ice, and used within 4 h. To permeabilize the OM, the pelleted cells were resuspended in 10 mL 100 mM Tris/HCl containing 10 mM EDTA, pH 8.0, then pelleted and re-suspended in 100 mM Tris/HCl, pH 8.0, to obtain 1 mL of concentrated suspension (~2 × 10^11^ cells/mL).

Heat-inactivated cells were prepared by incubating the concentrated suspension at 95 °C for 10 min. To evaluate the antimicrobial activity of the compounds, we applied the broth dilution method. The procedure involves serial two-fold dilutions of the antimicrobial compounds in the LB growth medium in 96-well microtitration plates. Each well was inoculated with a microbial inoculum where the concentration of bacteria cells was 5 × 10^5^ cfu/mL [22]. Microplates were incubated without agitation at 37 °C. The turbidity (λ = 612 nm) of the cell suspensions was measured using a “TECAN GENios Pro™” (Männedorf, Switzerland) plate reader after 16–20 h of incubation.

### 2.3. Electrochemical Measurements

A PAβN sensor was developed using electroactive material–ion–association complexes of PAβN cation with tungstosilicate anion dispersed in a PVC matrix. The membrane of the sensor was prepared following a well-known procedure [23] by dissolving poly(vinyl) chloride (PVC), dioctyl phthalate plasticizer, and tungstosilicic acid hydrate under stirring in freshly distilled tetrahydrofuran. The membrane components were taken in the following proportion (by weight): tungstosilicic acid hydrate, 1%; dioctyl phthalate, 66%; and PVC, 33%. The membrane was fixed to the sensor body, soaked overnight in 1 mM PAβN solution and washed in deionized water before the experiments. The sensors were stored dry at room temperature. Assembling the PAβN-selective electrode, the sensor was filled with 0.1 mM PAβN solution in 100 mM NaCl and connected to an internal Ag/AgCl reference electrode.

PAβN^+^ and TPP^+^-selective electrodes were connected to the potential-amplifying system based on an ultralow input bias current operational amplifier AD549JH (Analog Devices, Norwood, MA, USA) [21]. The amplifying system was connected to a computer through PowerLab 4/35 logger (ADInstruments Pty Ltd., Bella Vista, Australia). Ag/AgCl reference electrodes (model Orion 9001, Thermo Inc., Cambridge, MA, USA) were indirectly connected to the measuring vessels by agar salt bridges. Potentiometric measurements of the PAβN^+^ and TPP^+^ concentration in the incubation medium were performed simultaneously in two to three thermostated reaction vessels. The experiments were performed at 37 °C in 5 mL of magnetically stirred 100 mM Tris/HCl, pH 8.0, and TPP^+^ ions to 3 μM concentration were added to the cuvette. The concentrated cell suspension was added to obtain OD_600_ of 1. After adding the cells, the change in TPP^+^ concentration in the medium was registered due to the accumulation of these cations in the cells. Efflux pumps are non-selective, and they extrude different lipophilic compounds—antibiotics, dyes, etc. TPP^+^ is known as a universal efflux pump substrate and according to its accumulation in the cells, the efflux pump activity can be determined using a potentiometric system and TPP^+^-selective electrode. A representative set of curves from three independent experiments is presented.

## 3. Results

### 3.1. Inhibitory Efficiency of PAβN Depends on the Concentration of Treated Cells

**Figure 1:**
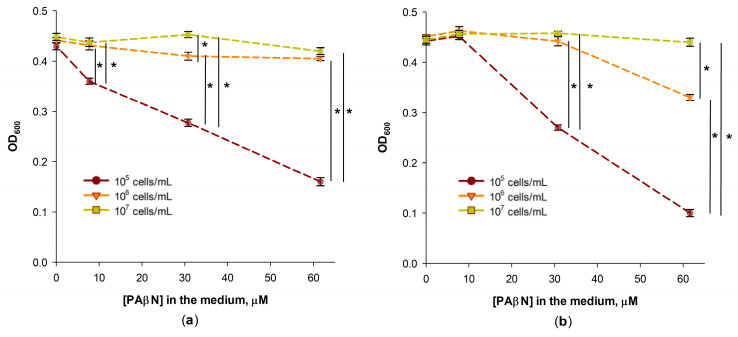
Effect of the initial concentration of *S.* Typhimurium cells on their growth in chloramphenicol (**a**) or tetracycline (**b**) containing LB medium in the presence of PAβN. The antibiotics were added to the final concentration of 0.25 mg/L, and the initial concentrations of cells are indicated in the figure. The cells were grown overnight in 96-well plates at 37 °C. Three biological replicates were performed for each set of experiments; *—*p* < 0.05.

In determining the minimal inhibitory concentration (MIC) of antimicrobials, it is important to select a proper initial concentration of the tested bacteria [22]. The MICs of chloramphenicol and tetracycline on *S.* Typhimurium cells grown in the LB medium were 4 and 2 mg/L, correspondingly, when the initial cell concentration was 10^5^ cfu/mL. Considerably lower, at 0.25 mg/L, concentrations of chloramphenicol and tetracycline affected the growth of *S.* Typhimurium cells when 60 μM of PAβN was present in the medium at the initial cell concentration of 10^5^ cfu/mL (Figure 1a,b). However, PAβN did not stimulate the inhibitory action of chloramphenicol when the initial concentration of *S.* Typhimurium cells was higher—10^6^ or 10^7^ cfu/mL (Figure 1a). PAβN had some stimulatory effect on the action of tetracycline when the concentration of *S.* Typhimurium was 10^6^ cfu/mL but had no effect on the efficiency of this antibiotic when the initial concentration of bacteria was 10^7^ cfu/mL (Figure 1b). The decreased efficiency of PAβN at higher concentrations of cells can be expected if PAβN bind to bacteria and this inhibitor is distributed to a 10- or 100-times higher number of cells.

### 3.2. At Low PAβN Concentrations TPP^+^ Is not Able to Penetrate the Cells

**Figure A1:**
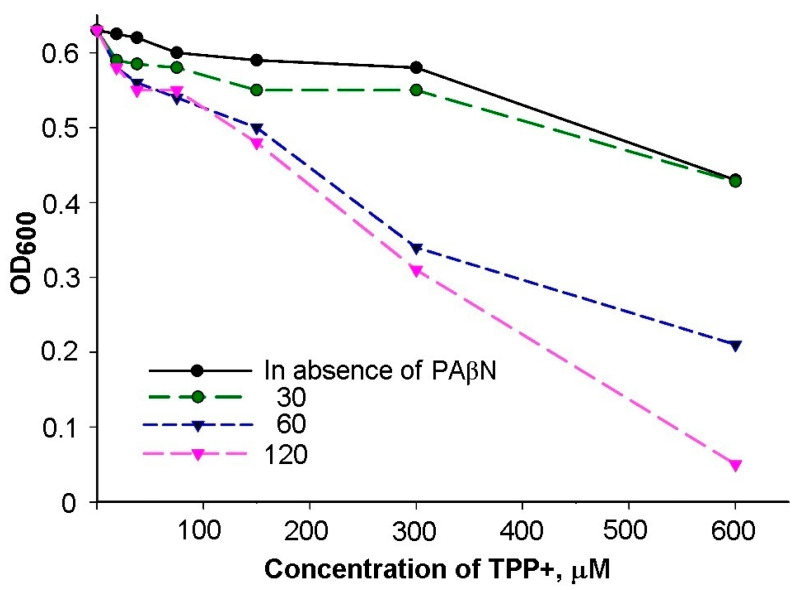
Effect of PAβN on the growth of *S.* Typhimurium cells in TPP^+^-containing LB medium. Serial dilutions of TPP^+^ were performed in the presence of the figure-indicated supplements of PAβN (μM). The cells were grown overnight in 96-well plates at 37 °C.

The results of the TPP^+^ microdilution test showed that PAβN can be characterized by some threshold values on TPP^+^ action on the cells. A considerable bacteriostatic effect of TPP^+^ on the growth of *S.* Typhimurium wt cells was achieved in the presence of 60 μM and higher concentrations of PAβN (Figure A1). Determining the effects of PAβN on the efficiency of antimicrobials using the microdilution method, we registered the efficiency of this efflux inhibitor on the growth of cells after 16–24 h of incubation in the LB medium.

**Figure 2:**
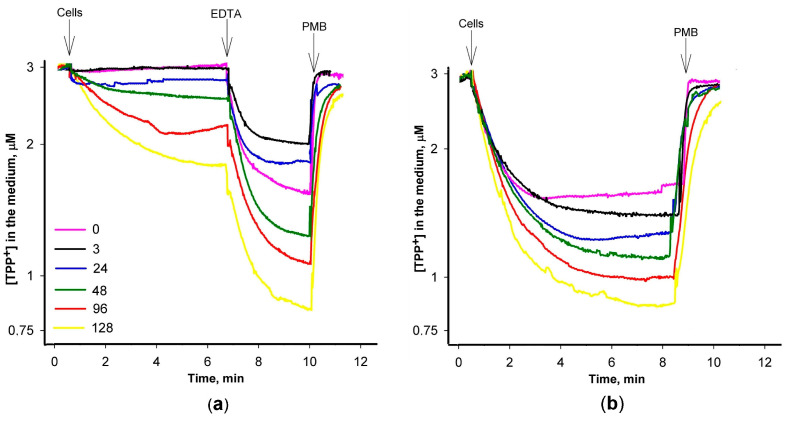
Influence of PAβN concentration on TPP^+^ accumulation in *S.* Typhimurium wt cells with (**a**) intact and (**b**) Tris/EDTA-permeabilized OM. The experiments were performed at 37 °C in 100 mM Tris/HCl buffer, pH 8.0, containing 0.1% of glucose. The cells were added to obtain the suspension OD_600_ of 1, EDTA (**a**)*—*to the final concentration of 1 mM, and Polymyxin B (PMB)*—*to 100 mg/L. PAβN was added to the medium before the cells to concentrations indicated in the figure (μM).

In the subsequent series of experiments, we delved into the impact of varying PAβN concentrations on the accumulation of TPP^+^ within *S.* Typhimurium cells using an ion-selective electrode, enabling the monitoring of the concentration of this lipophilic cation in the incubation medium (Figure 2). Cells with an intact OM exhibited a discernible TPP^+^ accumulation when exposed to PAβN concentrations exceeding 24 μM (Figure 2a). In contrast, a distinct and more pronounced accumulation of TPP^+^ by the cells was observed when Tris/EDTA-permeabilized cells were studied (Figure 2b). Even at a considerably lower PAβN concentration of 3 μM, these cells exhibited an augmented TPP^+^ accumulation (Figure 2b). The supplementation of EDTA to the medium induced an additional uptake of TPP^+^ by the cells with an intact OM (Figure 2a). In both cases, the increasing concentration of the efflux inhibitor in the medium resulted in a proportional increase in TPP^+^ uptake.

**Figure A2:**
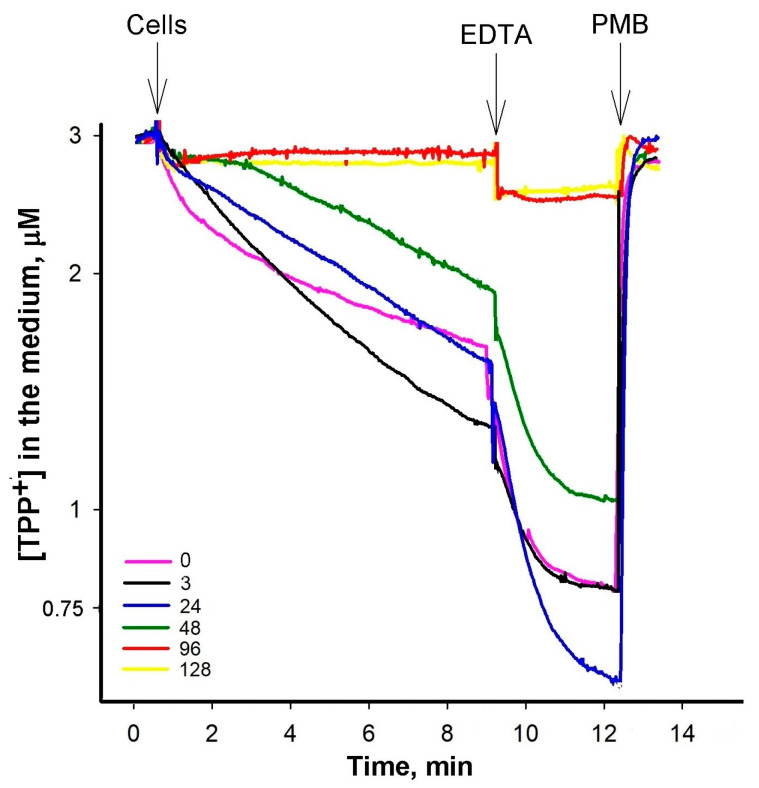
Influence of PAβN concentration on TPP^+^ binding to *S*. Typhimurium of ΔacrB mutant cells with intact OM. The experiments were performed at 37 °C in 100 mM Tris/HCl buffer, pH 8.0, containing 0.1% of glucose. The cells were added to obtain OD_600_ of 1, EDTA—to the final concentration of 1 mM, and Polymyxin B (PMB)—to 100 μg/mL. PAβN was added to the medium before the cells to concentrations indicated in the figure (μM).

**Figure A3:**
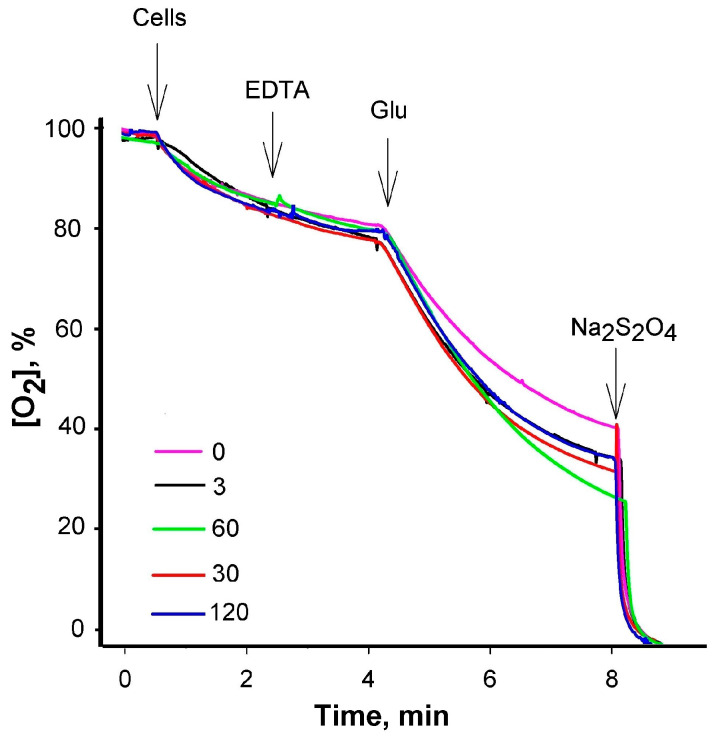
Effect of PAβN on the respiration of *S*. Typhimurium cells. Measurements of the dissolved oxygen concentrations in the medium were performed at 37 °C in 100 mM Tris/HCl buffer, pH 8.0, *S*. Typhimurium wt cells were added to OD_600_ of 1. The final concentrations of the supplements were as follows: EDTA—0.1 mM, glucose (Glu)—0.1%, and Na_2_S_2_O_4_—2 mg/mL. The concentrations (μM) of PAβN added to the medium before the cells are indicated in the figure.

However, the effect of the increasing PAβN concentrations was different when ΔacrB mutant cells were analyzed (Figure A2). The initial uptake of TPP^+^ was the highest at 3 μM PAβN. When the OM of ΔacrB cells was permeabilized by adding EDTA, the maximal TPP^+^ uptake was achieved in the presence of 24 μM PAβN. The following increase in PAβN concentrations in the medium resulted in a lower accumulation of TPP^+^ by the cells and in the presence of 96 μM PaβN, the TPP^+^ accumulation in ΔacrB cells was suppressed. An assessment of the PAβN effect on the medium-dissolved oxygen concentration showed that this efflux inhibitor did not affect the respiration rate and, most probably, the Δψ of *S.* Typhimurium cells at concentrations ranging from 3 to 120 μM (see Figure A3).

### 3.3. Most of PAβN Is Bound to the Cell Surface

**Figure 3:**
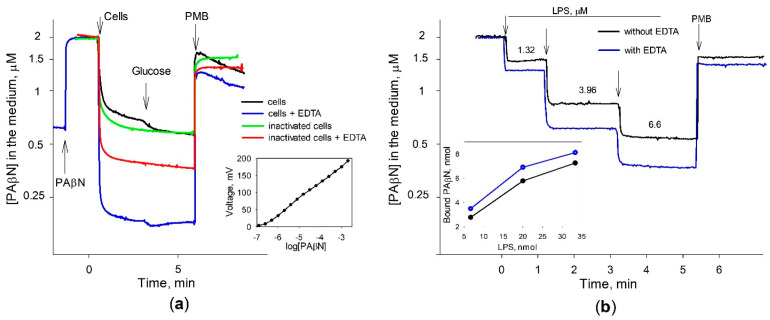
Binding of PAβN to *S.* Typhimurium cells (**a**) and LPS (**b**). The experiments were performed in 5 mL of 100 mM Tris-HCl, pH 8.0, at 37 °C. In (**a**) PAβN was added to the final concentration of 2 μM (Nerstian slope of the electrode is presented in the insert). The concentrated cell suspension was added to obtain OD_600_ of 1. Glucose and Polymyxin B (PMB) were added to the final concentrations of 0.1% and 50 mg/L, correspondingly. In (**b**) LPS additions are shown by the arrows. Numbers indicate the final concentrations of LPS after each of the additions. PMB was added to the final concentration of 50 mg/L, EDTA*—*to 0.1 mM. Insert shows the relationship between the amount of LPS added and the amount of PAβN bound.

To learn more about the mode of interaction between *S*. Typhimurium cells and the efflux inhibitor, a PAβN-selective electrode was developed. It allowed us to monitor 0.2 μM and higher concentrations of PAβN (see Figure 3a, insert). The electrode showed a stable PAβN concentration-dependent potential and near Nernstian response, approximately 56.5 +/− 0.5 mV/decade at 37 °C. The tested components of microbiological media and buffers did not affect the potential of the electrode and its response time was 10–15 s.

An addition of the intact *S*. Typhimurium wt cells to the inhibitor-containing 100 mM of Tris buffer induced a strong decrease in PAβN concentrations in the medium. In the EDTA-containing Tris buffer, *S*. Typhimurium cells bound considerably higher amounts of PAβN (Figure 3a). A supplement of glucose to the incubation medium caused a negligible additional binding of PAβN to the cells. Polycationic antibiotic Polymyxin B induced a sharp and very strong increase in PAβN concentrations in the medium, but just after that, the medium concentration of this inhibitor started to decrease. Heat-inactivated cells bound lower amounts of PAβN compared to the intact cells and the addition of glucose did not have any effect. The inactivated cells also bound lower amounts of PAβN in the presence of EDTA and the addition of PMB induced a sharp release of this inhibitor. It should be mentioned that after the PMB addition into the heat-inactivated cell suspensions, PAβN concentrations in the medium increased and remained stable. Another interesting observation is that some amount of PAβN remains bound to the cells in the presence of PMB, which is proportional to the maximal cell-bound amount at different experimental conditions.

The binding of PAβN to heat-inactivated cells and the sharp release of the cell-accumulated inhibitor after the addition of PMB suggest that PAβN binds to the cell surface. *S*. Typhimurium LPS shares an identical lipid A structure with that of *E*. *coli* [24]. The results of the experiments with the commercial purified LPS from *E. coli* cells supported this anticipation (Figure 3b). The added LPS bound a rather high amount of PAβN and the addition of PMB caused an immediate release of the inhibitor back to the medium. The LPS-bound amount of PAβN was directly proportional to the amount of LPS added (see the insert of Figure 3b) and in an EDTA-containing medium, LPS bound a higher amount of PAβN.

### 3.4. Saturation in PAβN Binding to S. Typhimurium Cells Can Be Achieved

**Figure 4:**
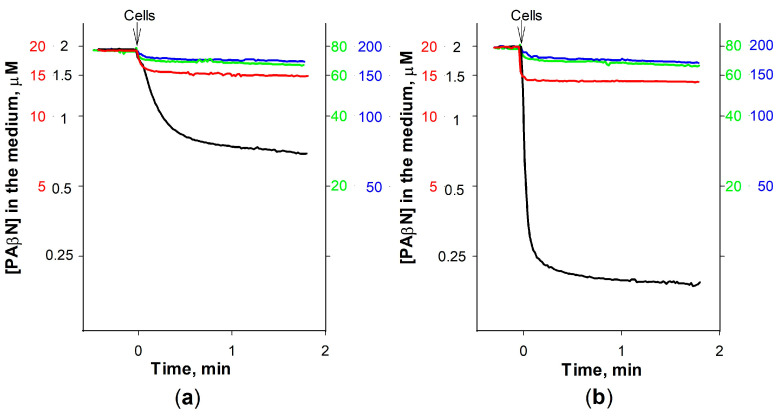
Effect of the initial concentration on PAβN binding to *S.* Typhimurium cells without EDTA (**a**) and in the medium contained 0.1 mM EDTA (**b**). The experiments were performed in 5 mL of 100 mM Tris-HCl, pH 8.0, at 37 °C. The initial PAβN concentration was 2 μM (black), 20 μM (red), 80 μM (green), or 200 μM (blue curves and scales). Arrows indicate the additions of cells.

To better understand the role of EDTA in PAβN interaction with the intact cells, the binding of this inhibitor to *S*. Typhimurium was studied using different PAβN concentrations. The best-pronounced electrode response indicated that relatively, the largest part of PAβN from the medium is accumulated by cells at the lowest PAβN concentration used (2 μM). A total of 1.5 × 10^10^ cells with an intact OM bound 6.5 nmol of PAβN and the same number of cells in the presence of EDTA—9 nmol of this inhibitor. At 20 μM of PAβN concentration, 1.5 × 10^10^ cells bound ~25 and ~30 nmol, correspondingly (Figure 4). At 80 μM and higher initial concentrations, the addition of cells caused a negligible change in the medium PAβN concentration and the impact of EDTA was also insignificant. According to our calculations, the maximal amount of PAβN that 1.5 × 10^10^ cells can bind is 25 or 30 nmol, in the absence or presence of EDTA, which is equal to the amount bound in the 20 μM solution of this inhibitor. For a single cell, this corresponds to 16.7–20 × 10^−19^ mol or ~1–1.2 × 10^6^ molecules of PAβN. The results obtained suggest that the high affinity of PAβN to bacterial LPS could be an important factor in strengthening its inhibitory action on efflux pumps in Gram-negative bacteria.

## 4. Discussion

Efflux pump inhibitors are considered to be promising tools to combat multidrug-resistant bacteria [6]. Considering the broad range of substrates, it is possible that bacterial efflux inhibitors would also affect the extrusion of lipophilic compounds from the eukaryotic cells. In this context, the knowledge of factors determining the selectivity of efflux inhibitors is of particular importance. PAβN is one of the best-known inhibitors of efflux pumps of the RND superfamily and by using a newly developed PAβN-selective electrode, we demonstrated that this compound with a high affinity binds to *S*. Typhimurium cells and LPSs from *E. coli*. The amount of PAβN bound to the LPS is directly proportional to the amount of LPS added into the solution of this efflux inhibitor, highlighting the specificity and concentration-dependent nature of this interaction.

Schuster et al. [25] showed that PAβN replaces Mg^2+^ in LPSs, and our experiments registered the PAβN binding increasing effect of EDTA, which is consistent with this observation. The stimulatory activity of EDTA on the accumulation of PAβN by the cells could be explained by the removal of divalent cations and the opening of the additional PAβN binding sites on the surface of the OM. The high density of negatively charged residues in LPSs is likely to be of physiological significance, as it concentrates bivalent cations such as Ca^2+^ and Mg^2+^ in the close environment of the cell surface where cations are required for the structural and functional integrity of the outer membrane [24]. The results of our experiments indicate that after the EDTA-induced destruction of the OM permeability barrier, the additional binding of PAβN to the cells is observed, indicating the modulatory role of EDTA in enhancing the binding affinity between PAβN and the LPS. At some experimental conditions, the massive binding of PAβN to the OM could be the reason for the observed permeabilization [26], but this is not an obligatory condition of the action of this efflux inhibitor [13].

The accumulation of PAβN in an anionic periplasm and a negatively charged cytosol could be the additional factors promoting the binding of PAβN to Gram-negative bacteria. Previously, we observed [14] that in an EDTA-containing medium, PAβN induces the efflux of TPP^+^ with a higher efficiency than from *S.* Typhimurium cells with an intact OM. On the other hand, a sharp PMB-induced release of PAβN, more prompt than the leakage of cell-accumulated TPP^+^, suggests that the accumulation of these cations in bacterial cells differs. First of all, supplementing the incubation medium with glucose induces only a negligible additional accumulation of PAβN, which is not followed by its partial extrusion, as is observed in the case of TPP^+^ [13,14]. These observations suggest that PAβN is not just another efflux pump substrate (competitive inhibitor) competing with TPP^+^ for the extrusion. According to Kinana et al. [7], PAβN inhibits the efflux of other drugs by binding to the bottom of the distal binding pocket (hydrophobic trap) and interferes with the binding of other drug substrates to the upper part of this pocket. Our data suggest that to get to the hydrophobic pocket, PAβN most probably does not need to cross the PM and enters into the pocket from the periplasm.

Efflux pumps may extrude PAβN from the periplasm, but it is possible that it is not released into the medium and stays bound to the outer layer of the OM. However, PMB, as a compound with a higher affinity to LPSs than PAβN, releases this inhibitor from the cell surface back to the medium. The immediate and very effective release of the bound PAβN upon the addition of PMB implies that PMB disrupts the PAβN–LPS bond, leading to the release of the bound inhibitor. The experimental results, captured in the insert of Figure 3b, offer a clear visual representation of this interplay.

The PAβN binding saturation effect is attained at high concentrations of this compound. The dimensions of *E. coli* and *S.* Typhimurium cells are very similar and one bacterial cell contains approximately 3.5 × 10^6^ of LPS molecules, occupying around three-quarters of the bacterial surface, with the remaining area being filled by proteins [27,28]. Our calculations indicate that one molecule of PAβN binds to ~3 LPS molecules on the cell surface. The results of the experiments with purified LPSs show very similar numbers.

Earlier, PAβN was known as a substrate for the colorimetric determination of peptidase activities. The incubation of intact *E. coli* cells with PAβN results in a time-dependent increase in fluorescence which has an emission spectrum corresponding to that of β-naphthylamine. Kinana et al. have shown [7] that the hydrolysis of PAβN in *E. coli* is nearly entirely dependent on an aminopeptidase, PepN, and the expression of this peptidase in the periplasm. We have found that the concentration of PAβN in the medium starts to decrease after the cell permeabilization with PMB. However, in the case of heat-treated cells, PMB treatment only leads to the release of PAβN back to the medium where the concentration of this compound stays stable.

If the molecular structures of TPP^+^ and PAβN are compared, the latter looks more hydrophilic, and a much higher OM penetration rate for TPP^+^ could be predicted. The Δψ-driven equilibrium distribution of PAβN between the cytosol and the incubation medium should be slower, but the binding of PAβN to cells as well as the release of this inhibitor from the cells to the medium after the cell treatment with PMB are very fast processes, supporting the idea that most of the PAβN does not cross the OM. Also, our results suggest that PAβN is not a proper efflux pump substrate compared to TPP^+^. Our earlier published results [13,14], along with the results of the experiments on the interaction of PAβN with the efflux pump mutant ΔacrB (Figure A2), show that at high concentrations, PAβN depolarizes the bacterial PM. However, the mechanism of this depolarization stays unclear if Δψ is not used for the massive efflux of this inhibitor. Although the information is limited, it is known that PAβN also affects the accumulation of antibiotics in Gram-positive bacteria [29,30] and mycobacteria [31]. These results indicate that efflux pumps from other families might also be sensitive to PAβN inhibition. It is possible that the experiments on PAβN binding to Gram-positive bacteria and mycobacteria would allow us to find out how important the accumulation of inhibitors on the cell surface is for the selective and effective blockage of efflux pumps.

## 5. Conclusions

The developed PAβN-selective electrode led us to the assessment of the *S*. Typhimurium cell affinity of this efflux inhibitor and provided valuable insights into the concentration-dependent dynamics of PAβN action on TPP^+^ accumulation, revealing different responses between intact and EDTA-permeabilized cells. This demonstrates the complex interplay of PAβN, and the outer membrane integrity and permeabilizing activity of EDTA, enriching our understanding of this bacterial efflux and its regulation. The electrode’s specificity was demonstrated in assessing the interaction of PAβN with *Salmonella enterica* ser. Typhimurium cells. Selective electrodes offer a potent means for real-time monitoring and quantification and enable the monitoring of PAβN at concentrations of 0.2 μM and higher, providing a rapid and stable response.

## Data Availability

The data presented in this study are available on request from the corresponding authors.
